# Unraveling Receptor Stoichiometry Using Bret

**DOI:** 10.3389/fendo.2012.00086

**Published:** 2012-07-12

**Authors:** James H. Felce, Simon J. Davis

**Affiliations:** ^1^T-cell Biology Group, Nuffield Department of Clinical Medicine, University of OxfordOxford, UK; ^2^MRC Human Immunology Unit, University of Oxford, John Radcliffe HospitalOxford, UK

The first and arguably most important question that could be asked about the biology of any protein is: does it function alone? Cell surface receptors present special problems for stoichiometric analysis because, being located within lipid bilayers, they are often very hydrophobic, which means that once isolated they can exhibit a strong tendency to aggregate. A very welcome development, therefore, has been the advent of *in situ* methods for probing receptor organization, the most important of which are presently based on resonance energy transfer. Our first bioluminescence resonance energy transfer (BRET) experiments were, however, inconclusive since both monomeric and dimeric receptors gave high levels of energy transfer (James et al., 2006). It was only with the application of theoretical principles first developed for (Fung and Stryer, 1978; Wolber and Hudson, 1979), and then used in (Kenworthy and Edidin, 1998), Förster resonance energy transfer (FRET) experiments that we could use BRET to confidently distinguish between monomers and dimers.

We were very keen to test G protein-coupled receptors (GPCRs) using the new approach given the great interest in these important proteins forming constitutive oligomeric complexes (Angers et al., 2000; Ramsay et al., 2002; Babcock et al., 2003). This seemed unlikely to us firstly because, structurally, GPCRs are ideally configured for functioning autonomously (Meng and Bourne, 2001) and, secondly, because functional autonomy explains the remarkable evolutionary success (Schiöth and Fredriksson, 2005) of this very large family of receptors. We were initially ignorant of the extent to which BRET was used to buttress the “GPCRs as oligomers” concept (Pfleger and Eidne, 2005), but when our initial analyses of human β_2_-adrenergic (β_2_AR) and mouse cannabinoid (mCannR2) receptors yielded the “BRET signatures” of monomers (James et al., 2006), we had to confront this body of data. The resulting controversy (Bouvier et al., 2007; James and Davis, 2007a,b; Salahpour and Masri, 2007) seems to have prompted the development of other, more complicated approaches. Here, we describe our experiences using BRET and briefly consider the merits of these alternative approaches.

## Once is not Enough

Like all resonance energy transfer-based methods, BRET is based on the principle of non-radiative energy transfer (Förster, 1948). In this case excitation energy is passed from a luminescent donor (luciferase) to a fluorescent acceptor protein, typically a modified variant of green fluorescent protein (GFP) such as yellow fluorescent protein or GFP^2^. Many early studies of surface receptors, particularly GPCRs, employed “conventional” BRET assays developed for analyzing interacting soluble proteins, in which donor- and acceptor-fused receptors are expressed at a single, fixed ratio, and BRET efficiency (BRET_eff_) is measured as relative to controls (Angers et al., 2000; Ramsay et al., 2002; Babcock et al., 2003). These early studies were largely unanimous in concluding that the receptors in question form homo- and hetero-oligomeric interactions and were significant in establishing the oligomeric GPCR paradigm (Pfleger and Eidne, 2005). We initially used this assay to determine whether an immune protein, CD80, forms dimers at the cell surface as implied by our crystal structure (Ikemizu et al., 2000), and were pleased to see strong energy transfer in our first experiments. However, the closely related protein, CD86, which is a monomer, also yielded high levels of energy transfer – as much as 25% of the levels obtained for covalent homodimers (James et al., 2006). We suspected that this was “background” energy transfer arising from random interactions within the membrane, a view strengthened by analysis of a second monomer, CD2. We concluded that conventional BRET assays could be problematic for measuring receptor organization in membranes because, within the crowded two-dimensional plane of the cell membrane, the signal arising from random interactions can reach significant levels.

## Theoretical Work-Arounds

**Figure 1:**
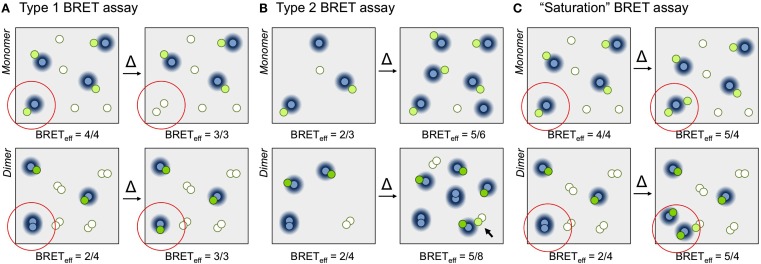
**Principles of BRET assays**. **(A)** In a type 1 BRET assay the acceptor/donor ratio is increased but surface density is kept constant. The increase in acceptor/donor ratio is obtained by exchanging a donor for an acceptor (the change is indicated within the red circle). For simplicity, BRET_eff_ is defined here as the ratio of the numbers of fluorescent acceptors and luminescent donors. In the examples shown, for the monomer (top) BRET_eff_ *is unchanged* (3/3 versus 4/4), whereas for the dimer (bottom) the ratio *increases* from 2/4 to 3/3 as the fraction of productive dimers increases. **(B)** In a type 2 BRET experiment, the acceptor/donor ratio is kept constant and surface density is varied, in this case by a factor of two. Due to the increase in monomer density (top) the likelihood of random collisions increases, and BRET_eff_ *increases* from 2/3 to 5/6. For constitutive dimers (bottom), however, BRET_eff_ is largely *unchanged*, increasing from 2/4 to 5/8, because the likelihood of dimerization doesn't change. Only random interactions of dimers (arrow) contribute to increases in BRET_eff_ (in reality, of course, these contributions will be significantly smaller than the effects of dimerization). **(C)** In the “saturation” BRET assay, the acceptor/donor ratio is increased by keeping donor numbers constant and increasing the numbers of acceptors. In the examples shown, BRET_eff_ for monomers (top) and dimers (bottom) *both increase* upon addition of one or two extra acceptors, respectively (from 4/4 to 5/4 for the monomer, and from 2/4 to 5/4 for the dimer). This is due to the increased random interactions of monomers, and increased formation and random interactions of dimers. We expect assays in which BRET_eff_ always increases to be more easily misinterpreted than assays in which changes in BRET_eff_ vary systematically with receptor stoichiometry.Fluorescing and non-fluorescing acceptor molecules are shown as green and white circles, respectively, and donors as blue circles. The BRET-permissible area surrounding donors is represented as a blue halo.

Theoretical considerations (Fung and Stryer, 1978; Wolber and Hudson, 1979; Kenworthy and Edidin, 1998) have predicted that the dependence of FRET on total and relative donor and acceptor concentrations differs systematically for specific and non-specific energy transfer. Applied to BRET in “type 1”experiments, total protein concentration is held constant and the acceptor/donor ratio increased by replacing donors with acceptors (Figure 1A; James et al., 2006). In this context, BRET_eff_ for monomers is independent of the acceptor/donor ratio above a certain threshold because donors always experience the same “acceptor environment.” For oligomers, however, replacing donors with acceptors reduces the fraction of donor–donor complexes, converting them into BRET-productive pairs and increasing BRET_eff_. In “type 2” experiments (Figure 1B; James et al., 2006) total protein density is varied at constant acceptor/donor ratio. For monomeric proteins BRET_eff_ varies linearly with total surface density for low expression levels, tending to zero at very low densities. Conversely, for constitutive oligomeric proteins BRET_eff_ is largely constant because expression itself is generally reliant on oligomerization. However, at high densities, BRET_eff_ increases due to random interactions of the oligomers within the membrane. For this reason it is inappropriate to draw any conclusions from the gradient of the slope for BRET_eff_ versus expression level as, e.g., in Ramsay et al. (2002).

Using these new types of BRET experiments we readily distinguished well-known monomeric and dimeric Type I membrane proteins, and even confirmed that CD80 forms apparently transient dimers at the cell surface, as implied by analytical ultracentrifugation (Ikemizu et al., 2000). Applied to two GPCRs, β_2_AR and mCannR2, these assays yielded the unambiguous “BRET signatures” of monomers (James et al., 2006). We also showed that the GABAβ receptor, a *bona fide* GPCR dimer, gave data characteristic of dimers and that transfer of the cytoplasmic domain of GABAβR2 to β_2_AR converted monomer-like into dimer-like behavior. As expected, since β_2_AR and other GPCRs were widely believed to form homo- and hetero-dimers (reviewed in Bouvier, 2001), these findings were controversial (Bouvier et al., 2007; James and Davis, 2007a,b; Salahpour and Masri, 2007).

## Alternative Assays

Broadly speaking there is now consensus that conventional, single-ratio BRET experiments are inadequate to the task of assigning receptor stoichiometry. However, although type 1 and 2 BRET and FRET experiments are done occasionally (e.g., Kenworthy and Edidin, 1998; Meyer et al., 2006), these approaches are not widely used. Instead, the so-called BRET “saturation” assay first used in 2002 (Figure 1C; Mercier et al., 2002) remains popular (Contento et al., 2008; Ayoub and Pfleger, 2010). In this approach, donor numbers are kept constant and acceptor expression systematically increased. Under such conditions BRET_eff_ for a monomeric protein is linearly related to acceptor expression level, whereas for oligomers the relationship is hyperbolic. The problem therefore becomes one of distinguishing between two increasing signals, which we would expect to be more difficult than distinguishing between increasing versus non-increasing signals, as in type 1 BRET assays (James et al., 2006). The problem becomes more acute for transient oligomers whose signals emerge from monomer/dimer equilibria, which is particularly relevant now that GPCRs are being claimed to transiently dimerize (Hern et al., 2010; Lambert, 2010; Kasai et al., 2011).

A second, newer assay, the “BRET competition” assay, presents subtler problems. In this assay, untagged “competitor” receptors are co-transfected with acceptor- and donor-tagged proteins, leading to reduced BRET_eff_ for oligomers and unchanged BRET_eff_ for monomers (Veatch and Stryer, 1977). In our experience, expression of untagged competitors often reduces expression of their tagged equivalents (Felce et al., unpublished data), including monomer control proteins, reducing BRET_eff_ artifactually. In BRET competition assays of GPCR homo- and heterodimerization (e.g., Terrillon et al., 2003; Guo et al., 2008), reduced energy transfer in the presence of untagged competitors is always observed, yet the issue of surface density is never addressed. Such approaches have their place but the absolute levels of tagged protein must be factored in to avoid ambiguity.

## Control Problems

An important factor complicating some BRET experiments is the heterogeneity of protein distribution, emphasizing the importance of the careful choice of controls. The cell membrane is a highly complex environment (Kusumi et al., 2011), and evidence is mounting that complex regulatory processes may control the localization and movement of integral membrane proteins, including GPCRs (Meyer et al., 2006; Nikolaev et al., 2010; Weigel et al., 2011). The potential for proteins to be localized to different areas of the cell surface, or to have different constraints on their trafficking, has important implications for data interpretation. This applies especially to “irrelevant” controls, which should have similar hydrodynamic diameter to the protein of interest but be sufficiently unrelated to not form specific associations (Angers et al., 2000; Mercier et al., 2002). However, such proteins may not be similarly localized at the membrane. For example, if the control protein exhibits strong association with the cytoskeleton but the protein of interest does not, BRET_eff_ will be lower in the control experiment than it would be if the two proteins co-localized but randomly interacted. Similarly, control proteins may be expressed at different total densities or have different stoichiometries, adding further complications. Without knowing their behavior and expression characteristics in detail, it is difficult to select appropriate controls.

Approaches in which acceptors are recruited to donor-tagged proteins of interest are especially dependent on control choice. In “Third-party BRET” (Kuravi et al., 2010), a membrane-associated acceptor is chemically recruited to an untagged receptor of interest and BRET_eff_ increases if the untagged receptor is a dimer that brings with it a donor-tagged receptor, the goal being to avoid the complication of varying expression levels. However, if the receptors are co-localized but do not interact, then acceptor/untagged receptor dimerization could recruit the acceptor to an area of greater donor concentration, increasing BRET_eff_ without genuine association. Similar arguments apply to GPCR-Heteromer Identification Technology (GPCR-HIT; Pfleger, 2009; Mustafa and Pfleger, 2011). For this reason, no conclusively reliable BRET-based assay for heterodimers presently exists. Despite these difficulties, conventional (Pfleger and Eidne, 2005), saturation (Sohy et al., 2009), and competition (Terrillon et al., 2003) BRET assays have all been used to support claims for GPCR heterodimerization.

## Concluding Remarks

There is now implicit agreement that single measurements of BRET_eff_ are unhelpful because the contribution of random interactions to the signal is not easily discerned. Similarly, the notion that varying expression levels can also give potentially misleading changes in BRET_eff_ is taking root, prompting new methods such as “Third-part BRET,” which seek to control for background effects in single measurements. The problem with these approaches is their heavy reliance on negative controls, which as we have discussed are often difficult to choose. We are surprised that the relatively simple approaches involving systematic variations of the acceptor/donor ratio, or of expression level alone, are not more widely used. We emphasize once again that the key to these methods is their exclusive reliance on the measurable, intrinsic behavior of populations of receptors diffusing in the plane of the membrane, and that an important advantage is that the assays are effectively control-independent.

Overall, the question of whether or not GPCRs generally form oligomers remains unsettled. The notion that they do is driven not only by BRET experiments, but also by FRET (Albizu et al., 2010; Cunningham et al., 2012), photon-counting analyses (Kilpatrick et al., 2012), and single-molecule microscopy (Hern et al., 2010; Kasai et al., 2011). We are seeking to test our BRET-based conclusions using super-resolution imaging, and to address GPCR stoichiometry at the family level using type 1 BRET and other experiments implemented in a high throughput setting.

Despite the controversies over its use BRET still has a very bright future. New luciferases, such as Rluc2 and Rluc8 (De et al., 2007), and acceptor fluorophores, such as Venus (Kocan et al., 2008), mOrange (De et al., 2009), and Renilla GFP (RGFP; Kamal et al., 2009), are brighter and offer up the possibility of *in vivo* studies (De et al., 2009). Future developments in BRET-quantum dot (Wu et al., 2011; Quiñones et al., 2012) and BRET-FRET (Carriba et al., 2008) assays will also advance the technique. The effective resolution of resonance energy transfer methods in live cells, i.e., ∼10 nm, is presently significantly better than that of *in situ* single-molecule imaging techniques, which, even in fixed cells, is limited to ∼20 nm (Moerner, 2012). We think that it will be some time before BRET, rigorously applied, is surpassed as a probe of receptor stoichiometry.
